# Supplementary figures and images for: Intracellular Cryptococcus neoformans disrupts the transcriptome profile of M1- and M2-polarized host macrophages
Source: PLoS One. 2020 Aug 28;15(8):e0233818. doi: 10.1371/journal.pone.0233818 (PMC7454990; doi:10.1371/journal.pone.0233818)

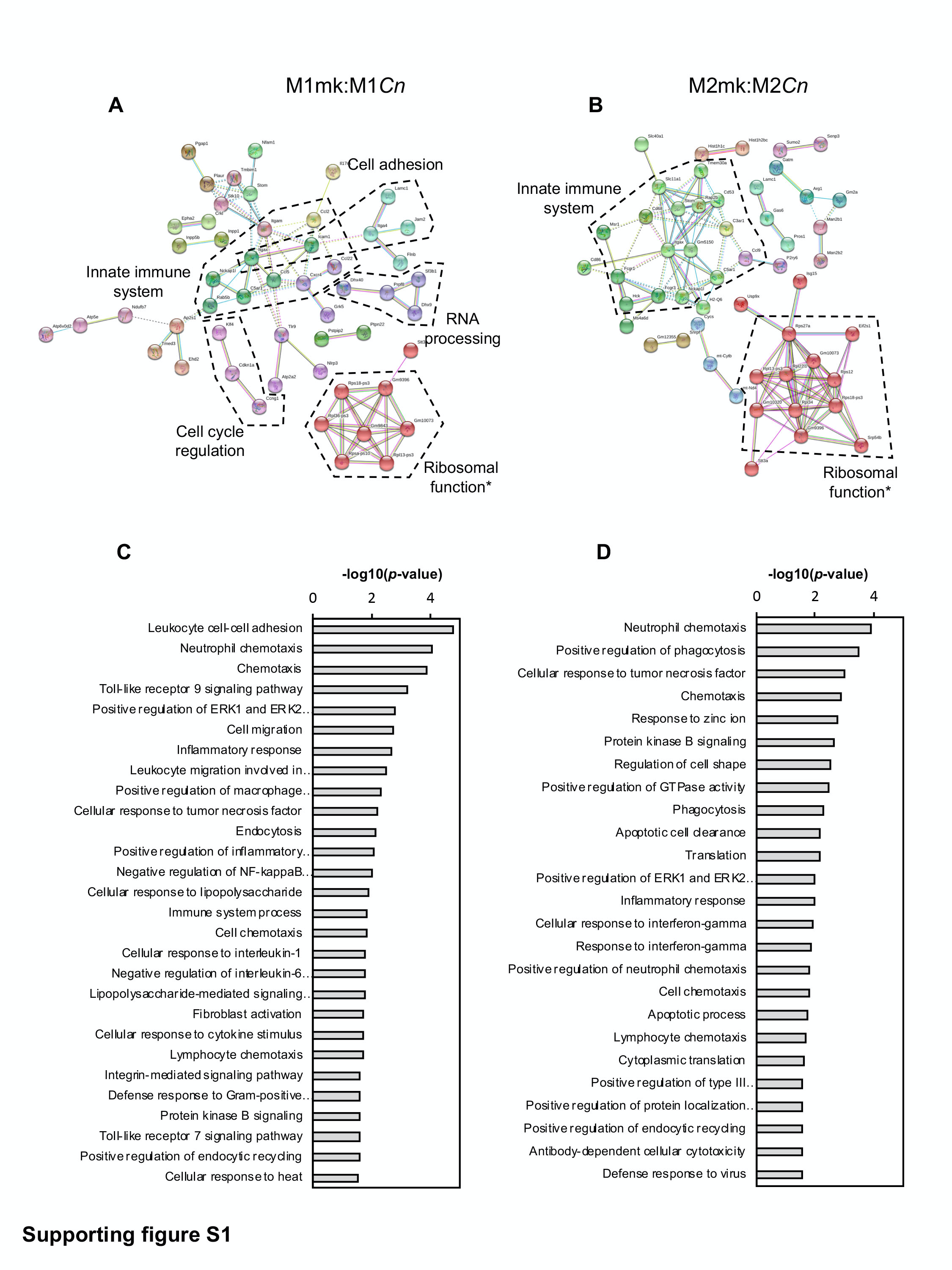

Supplement: S1 Fig — Boundaries enclosing gene clusters with common function were drawn based on gene GO term data and information from the literature. *Note: Many of the genes contained within the ‘Ribosome function’ boundary are pseudogenes. GO analysis performed in DAVID on DEGs from (S1C) M1mk:M1Cn and (S1D) M2mk:M2Cn pairwise comparisons. Relevant pathways are ranked by–log(p-value). (TIFF) [file pone.0233818.s001.tiff]

Uncropped images of the blots presented in the main figures

Fig. 1

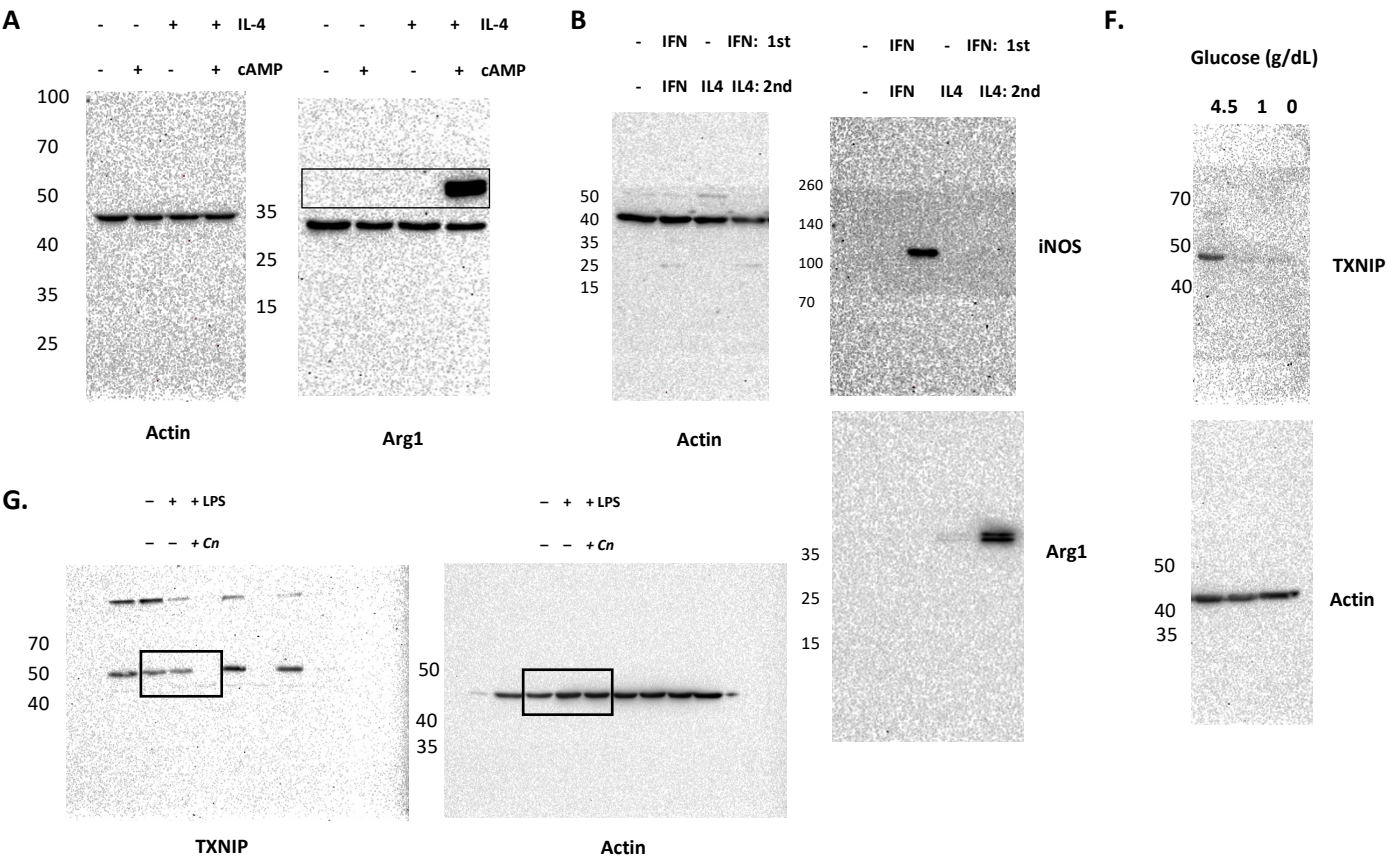

Fig.2

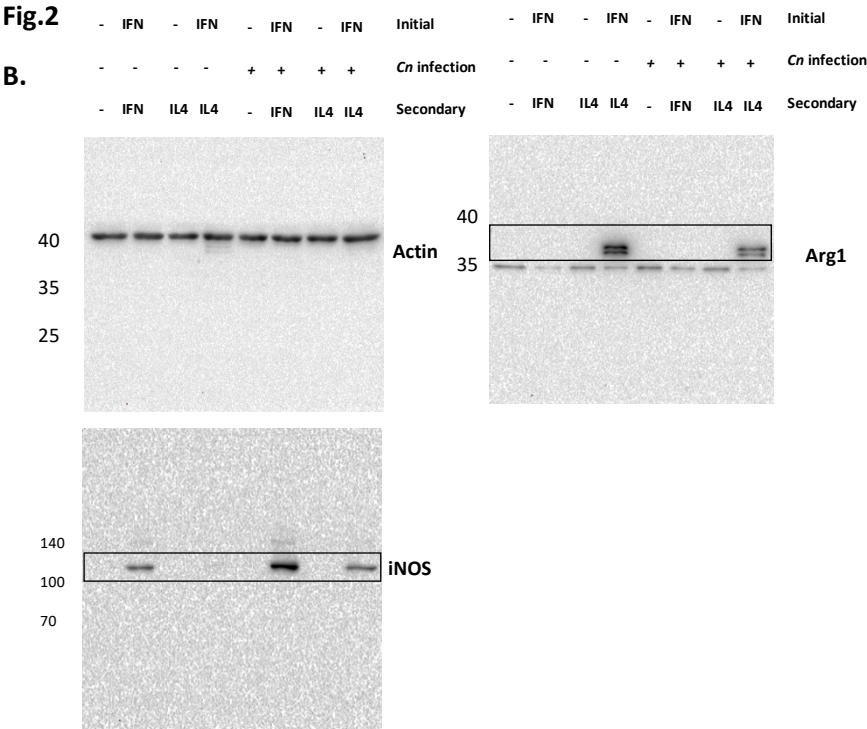

Fig. 5

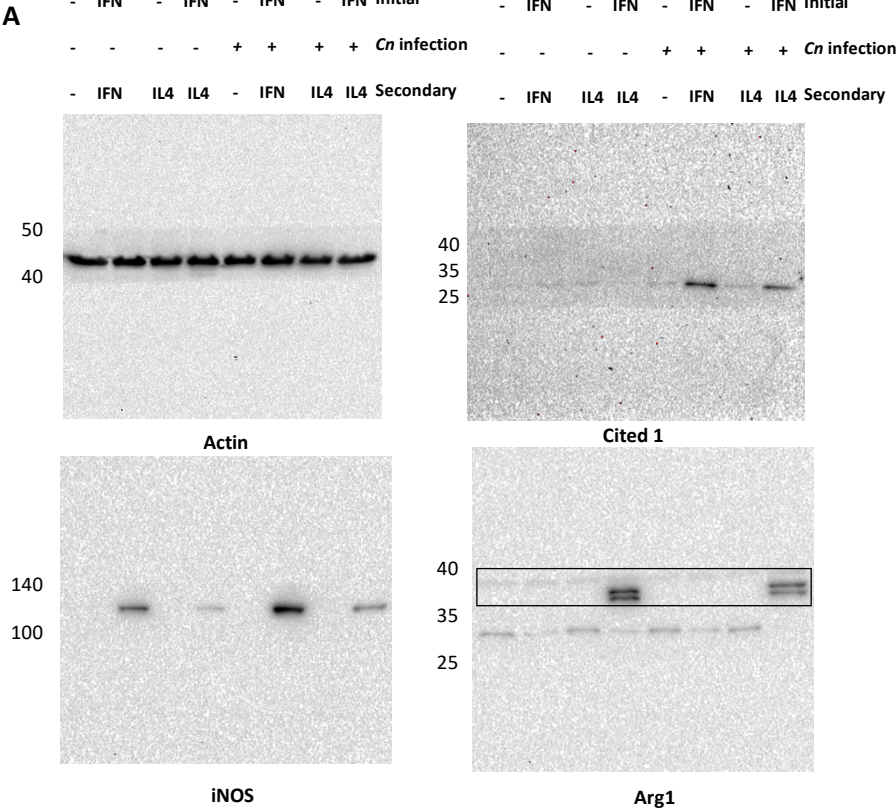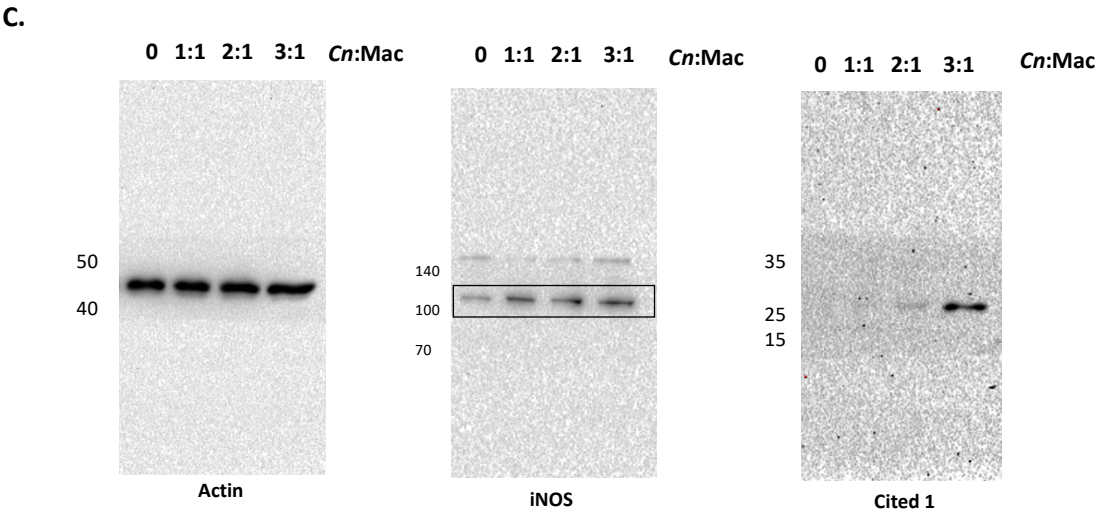

Supplement: S3 File — (PDF) [file pone.0233818.s004.pdf]
